# Hedgehog signaling: modulation of cancer properies and tumor mircroenvironment

**DOI:** 10.1186/s12943-016-0509-3

**Published:** 2016-03-18

**Authors:** Ann Hanna, Lalita A. Shevde

**Affiliations:** Department of Pathology and Comprehensive Cancer Center, The University of Alabama at Birmingham, Wallace Tumor Institute 320D, 1824 6th Avenue South, Birmingham, 35233 Alabama USA

**Keywords:** Cancer, Hedgehog signaling, Angiogenesis, Microenvironment, Epithelial-mesenchymal transition, Drug resistance, Metastasis, Cancer stem cells

## Abstract

Cancer poses a serious health problem in society and is increasingly surpassing cardiovascular disease as the leading cause of mortality in the United States. Current therapeutic strategies for cancer are extreme and harsh to patients and often have limited success; the danger of cancer is intensified as it metastasizes to secondary locations such as lung, bone, and liver, posing a dire threat to patient treatment and survival. Hedgehog signaling is an important pathway for normal development. Initially identified in *Drosophila*, the vertebrate and mammalian equivalent of the pathway has been studied extensively for its role in cancer development and progression. As this pathway regulates key target genes involved in development, its action also allows for the modulation of the microenvironment to prepare a tumor-suitable niche by manipulating tumor cell growth, differentiation, and immune regulation, thus creating an enabling environment for progression and metastasis. In this review, we will summarize recent scientific discoveries reporting the impact of the Hedgehog signaling pathway on the tumor initiation process and metastatic cascade, shedding light on the ability of the tumor to take over a mechanism crucially intended for development and normal function.

## Background

In order for tumor initiation and metastasis to occur, tumors usurp several signaling pathways that contribute to normal growth and tissue repair. The normal development of mammalian embryos involves multiple signaling pathways that regulate cell proliferation, survival, and differentiation such as the Hedgehog (Hh) [1], Wnt [2], TGF-β [3], and Notch [4] signaling pathways. When cells become cancerous, they recruit cellular machinery that typically promotes regular physiological functions such as tissue growth, survival, vascularization, and healing. Hh signaling is involved early in embryogenesis where it promotes cell growth, differentiation, tissue patterning, and vascularization; all of which are aspects that tumor cells employ in order to thrive and metastasize. In adult tissue, the activation of the Hh pathway maintains homeostasis by being limited to stem cell subsets that undergo rapid turnover and modulate tissue repair [5] such as the nervous system [6], skin [7] and intestines [8]. The Hh signaling pathway becomes aberrantly activated through the overexpression of Hh ligands, loss-of-function of the receptor, or dysregulation of the transcription factors. All these aberrations have been implicated in initiation and progression of multiple cancer types including breast, prostate, hepatocellular, pancreatic, and brain cancers. As such, the pathway has been intensely investigated for the development of inhibitors for therapeutic benefit in human cancers. In this review, we summarize recent advances on the influence of Hh signaling on tumor initiation, progression, and metastasis through modulating a multitude of processes that are considered signature features of cancer as were characterized by Hanahan and Weinberg [9, 10].

### The mammalian Hh signaling pathway

**Fig. 1:**
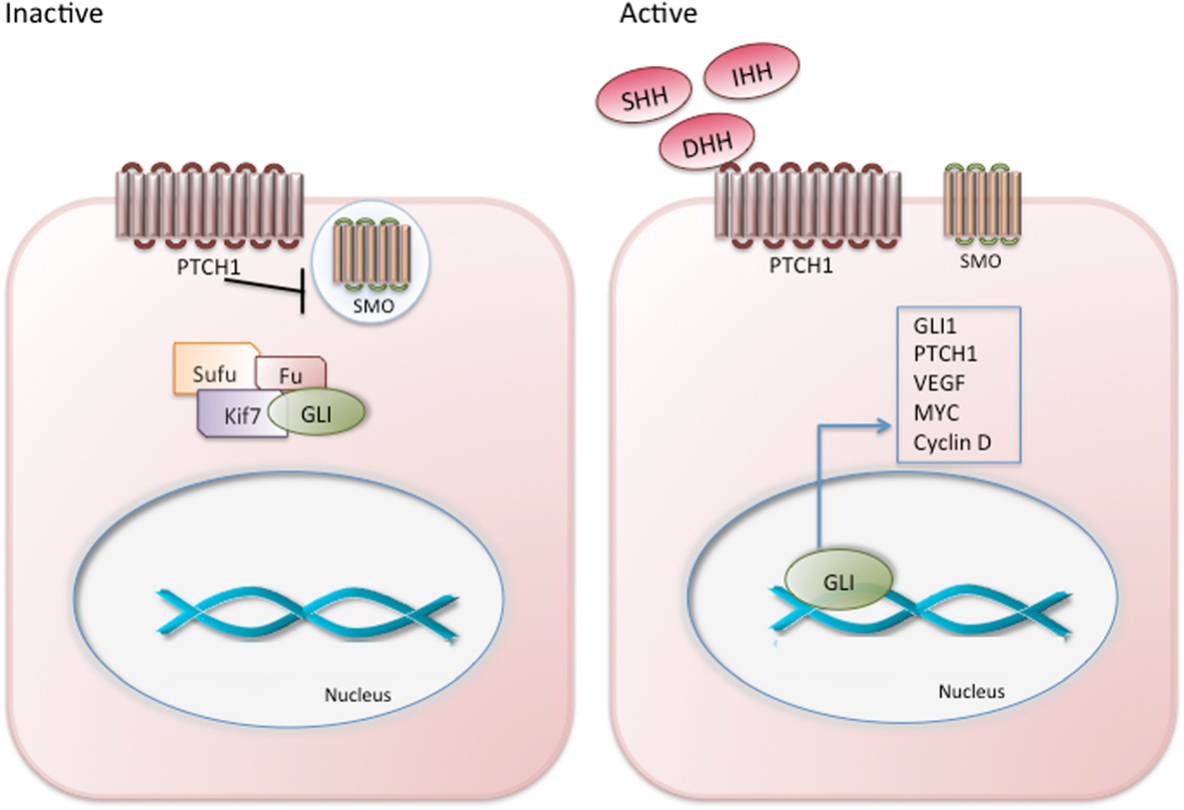
In the absence of Hh ligands, the PTCH receptor sequesters SMO in a cytoplasmic vesicle. GLI proteins are bound in a complex in the cytoplasm with negative regulators SUFU and KIF7, which target the GLI proteins for phosphorylation by PKA, CK1, and GSK3β inducing the repressive form of GLI proteins. Upon DHH, IHH, or SHH binding to PTCH, PTCH relieves its inhibition on SMO, which then translocates to the primary cilium of the cell and mediates the dissociation of GLI proteins from SUFU and KIF7, allowing localization of GLI proteins to the nucleus where they bind DNA and regulate the transcription of their target genes

Hh signaling involves a complex network of molecules. The canonical Hh signaling pathway can be initiated by three ligands: Desert hedgehog (DHH), Indian hedgehog (IHH) or Sonic hedgehog (SHH). These bind to the 12-pass transmembrane protein receptors Patched1 (PTCH1) and Patched 2 (PTCH2), which were initially found to form a complex with single pass co-receptor proteins: Cell adhesion molecule-related/downregulated by oncogenes (CDON) and Brother of Cdo (BOC) [11, 12]. The glycosylphosphatidylinisotol (GPI) anchor receptor Growth arrest-specific 1(GAS1) completes this co-receptor complex [1]. Once ligand binding occurs, PTCH receptors relieve their inhibitory action on Smoothened (SMO), a 7-pass transmembrane G-protein coupled signal transduction molecule, which then activates a signaling cascade resulting in the translocation of the glioma associated oncogene homolog (GLI) transcription factors to the nucleus. Emerging evidence suggests Hh ligand binding to each of the co-receptors, not only to PTCH as initially thought, is obligatory for the activation of the Hh signaling cascade [13]. In the absence of the ligand, SMO is normally localized in vesicles. When the pathway is activated, SMO localizes to the primary cilium on the cell membrane (Fig. 1). There are three GLI transcription factors: GLI1 that exclusively acts as a transcription activator, GLI2 and GLI3 that can act either as repressors or activators, in a context-dependent manner [14]. In their full-length form, all three transcription factors can be activators. GLIs 2 and 3 however can be processed into their truncated repressive form. Protein kinase A (PKA) is the molecule responsible for the activation or repression of the GLI proteins depending on the specific sites of protein it phosphorylates [15]. GLI3 acts as the main repressor of the pathway in the absence of Hh ligands, whereas, in their presence, the main activator GLI2 leads to the activation of GLI1, promoting its translocation into the nucleus where it initiates the transcription of downstream target genes.

In the nucleus, the GLI transcription factors bind DNA and modulate the transcription of a multitude of genes including Fox, Myc, and cyclin D among others that are involved in tissue development, differentiation, epithelial-mesenchymal transition (EMT), and stem cell maintenance. Recently, GLI1 protein transcript was found to undergo alternative splicing establishing two isoforms with different functions: an N-terminal deletion and a truncated GLI1 variant [16]. The truncated isoform is found only in cancer tissue and promotes transcription of additional target genes that create a pro-tumorigenic environment for tumor cells to expand.

Although the Hh signaling pathway is normally activated upon ligand binding to the receptor, multiple mechanisms have been involved in enhancing GLI protein activity in the absence of Hh ligands. In breast cancer, TGF-β signaling was shown to activate GLI2 target genes culminating in enhanced bone metastasis [17]. In basal cell carcinoma (BCC), mutated PTCH1 expression prevented cellular response to cell cycle checkpoint cyclin B1 and promoted GLI activation [18]. In Burkitt lymphoma, oncogene c-MYC was found to regulate GLI1 expression independently of SMO, PTCH or the presence of Hh ligands [19]. Furthermore, several reports in a pancreatic cancer model confirm that Hh pathway is activated in a paracrine manner [20]. Other signaling pathways have also been demonstrated to directly activate GLI proteins in a variety of cancer types such as PI3K/AKT [21–23] and RAS/ERK [24, 25] signaling have also been demonstrated to directly activate GLI proteins. Therefore, it is clear that the activation of GLI proteins and the transcription of their downstream target genes are not restricted to classical Hh signaling which occurs through ligand binding to the PTCH receptor and co-receptors, but can proceed *non-classically* as a result of crosstalk between diverse intracellular signaling networks.

### Hh pathway mechanisms of activation

The Hh signaling pathway must be strictly controlled in order to prevent aberrant activation. The Hh signaling pathway can be activated by four different mechanisms. And though the downstream effects of Hh activation are ultimately proliferation and differentiation, it is notable that its mode of activation differs depending on the cancer type [26, 27].

The Hh signaling pathway can be activated classically through ligand binding or non-classically in the absence of the ligand [28], both of which are GLI-dependent. Canonical, classical activation of Hh signaling involves the presence of Hh ligands, which initiate the signaling cascade leading to GLI translocation to the nucleus and binding DNA; this can occur as autocrine, paracrine, or inverse paracrine activation. Autocrine Hh signaling depends on tumor cells secreting Hh ligands, which then act upon themselves in a positive feedback loop; this mode of activation is found in breast cancer [29], non-small cell lung cancer [30], and colorectal cancer [31]. Paracrine signaling, on the other hand, comprises of tumor cells secreting Hh ligands that bind receptors on the surrounding stroma thus activating stromal Hh signaling; this is mainly found in pancreatic cancers [32, 33]. In inverse paracrine Hh activation, however, stromal cells produce the Hh ligands, which bind and activate the signaling pathway in tumor cells; multiple myeloma [34] and lymphoma [35] display this mode of activation.

The non-classical mode of activation is, as mentioned above, independent of Hh ligand presence, which occurs irrespective of PTCH receptors loss of function or gain of function of SMO; this mode is found in BCC [36], and can occur in breast cancer [37]. Alternative signaling pathways such as PI3K [21–23] and RAS [24, 25] signaling are also capable of activating non-classical Hh signaling by direct binding and activation of the GLI proteins. The pathway can also be activated non-canonically, which is independent of GLI activation [38]. Non-canonical Hh signaling is the result of upregulation of genes involved in the initial steps of the pathway such as SMO upregulation by SDF-1 in pancreatic cancer [39]. Hh ligand and SMO are also capable of inducing angiogenesis and reducing apoptosis in epithelial cells [40] by directly interacting with GTPase RhoA in the absence of GLI. Additionally, mutations that result in overproduction of Hh ligands in breast cancer [41] and BCC [42], loss of PTCH receptor function in BCC [43] and gastric cancer [44], as well as upregulation of SMO activity in pancreatic cancer stroma [45] and BCC [46] can lead to constitutively activated Hh signaling [47].

### Targeting the Hh pathway components

The first identified naturally occurring Hh blocker is cyclopamine, which causes developmental abnormalities in animals. The Beachy group first reported the inhibitory effect of cyclopamine on Hh signaling to occur by directly binding and inhibiting SMO [48]. However, the weak potency of the natural chemical elicited the need to develop modified chemical derivatives, which spurred the development of KAAD-cyclopamine [49]. Aside from SMO inhibition, several other Hh inhibitors have been identified for use in basic research. Small molecule inhibitors, GANT-58 and GANT-61 prevent GLI proteins from binding to DNA in the nucleus, thus blocking their transcriptional activity [50]. Additionally, the monoclonal antibody 5E1 is used in research to block SHH, IHH, and DHH binding to the PTCH1 receptor thereby, blocking the classical Hh signaling pathway [51].

The first FDA approved Hh inhibitor for clinical use is vismodegib (Genentech) marketed as Erivedge [52]. Approved in 2012, vismodegib targets SMO and is used to treat BCC patients, with promising results in other cancer types. Another SMO antagonist erismodegib (Novartis), also known as sonidegib and LDE-225, was recently FDA approved for treating BCC patients with recurrent disease or for those do not qualify for radiation or surgical removal [53].

Currently, there are several Hh inhibitors employed in clinical trials for multiple types of cancers. For example, sonidegib is being tested on patients with advanced hepatocellular carcinoma [54] and in combination with Paclitaxel in patients with recurring ovarian cancer [55], Bortezomib (a proteasome inhibitor) in patients with multiple myeloma (MM) in a recently closed trial [56], and with Docetaxel for patients with triple negative breast cancer [57]. Another SMO antagonist glasdegib, also known as PF-04449913, is being tested in patients with acute myeloid leukemia (AML) [58] and in combination with nucleoside analog Azacitidine for other hematologic malignancies such as chronic myelomonocytic leukemia [59]. Although Hh inhibitors have been mostly promising, several clinical trials have ended in failure. In 2012, Infinity Pharmaceuticals terminated a phase II clinical trial in pancreatic cancer patients employing SMO inhibitor saridegib in combination with gemcitabine as patients treated with this combination had an overall lower survival than the placebo treated group [60]. Another clinical trial in metastatic pancreatic cancer testing the combination of vismodegib with gemcitabine showed no significant differences in Hh pathway expression in the combination vs gemcitabine alone treatment [61]. In metastatic colorectal cancer, patients treated with vismodegib in combination with bevacizumab and FOLFOX or FOLFIRI (standard of care for metastatic colorectal cancer), did not add significant benefits to patient survival [62] Although Hh inhibitors effectively treat BCC, for which they were initially approved, their usage in treating other tumor types potentially carries significant risks, therefore, the mechanisms of Hh signaling should be critically evaluated in specific tumor types prior to commencing Hh inhibitor clinical trials in patients.

**Fig. 2:**
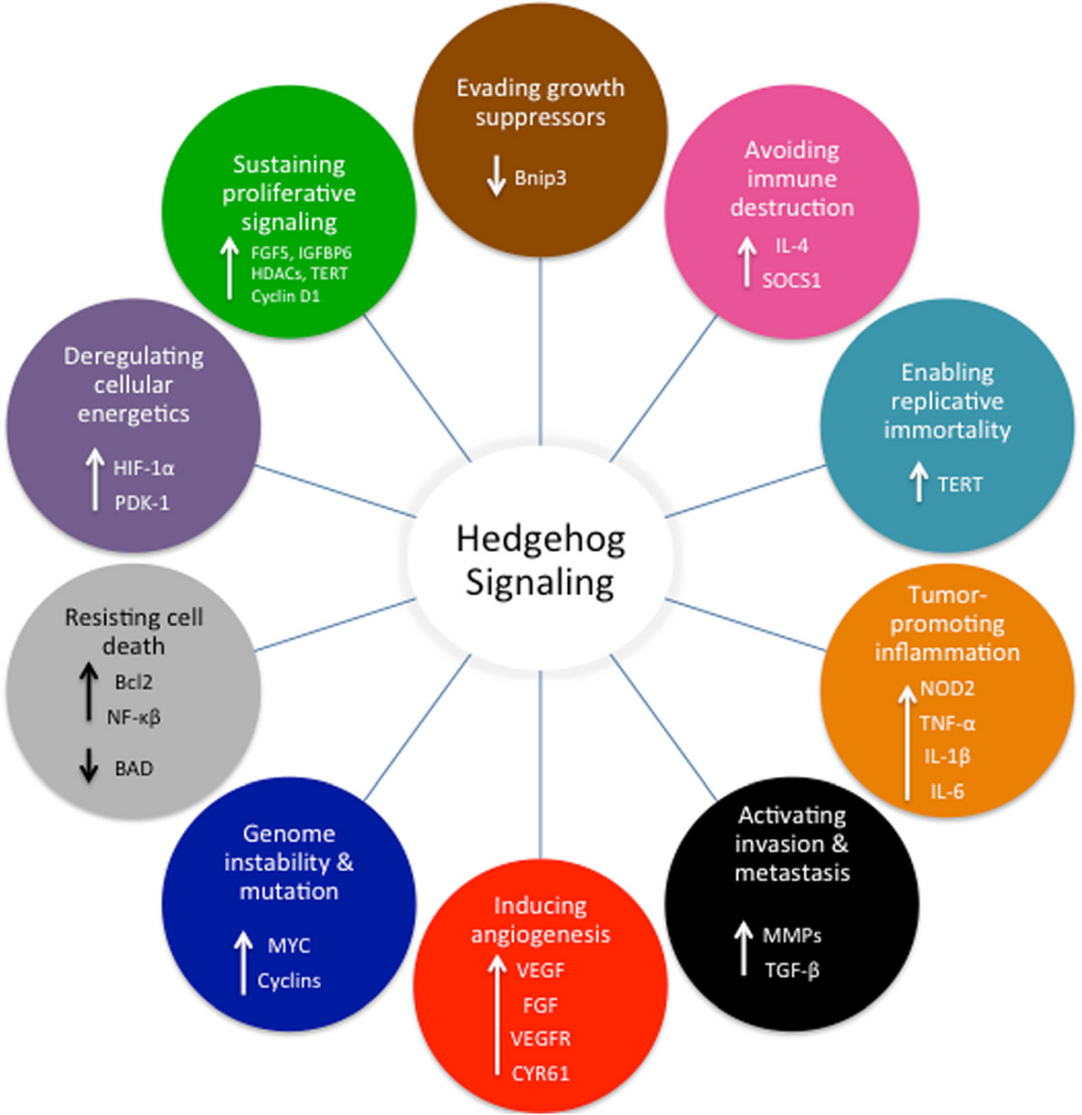
The Hedgehog signaling pathway plays an important role in tumor initiation and metastasis through profoundly altering the ten hallmarks of cancer

In the ensuing paragraphs, we will summarize recent scientific discoveries reporting the impact of the Hh signaling pathway on the tumor initiation process and metastatic cascade, shedding light on the ability of tumors to takeover a mechanism crucially intended for development and normal function. We have specifically focused on the recent advances and knowledge garnered to address novel tumor properties that pertain to creating a favorable microenvironment known as the updated hallmarks of cancer as described by Hanahan and Weinberg (Fig. 2).

### Sustained proliferative signaling

The most pronounced characteristic of cancer is the unrestricted proliferation of cells. As the main function of Hh signaling is to promote fetal development and patterning, it is expected that signaling through the pathway would upregulate genes involved in cellular proliferation and survival, a function that tumors manipulate. Not only does Hh signaling promote the over-proliferation of cancer cells themselves, but it also modulates cells found in the local microenvironment. Wilkinson and colleagues found that human prostate cancer associated fibroblasts (CAFs) have the capability of forming primary cilia; this was associated with upregulation of canonical Hh signaling [63]. Additionally, engraftment of non-malignant prostate cells bearing constitutively active Smo into mice resulted in increased levels of cellular proliferation by the prostate cells. The enhanced proliferation of cells was attributed to the expression of increased fibroblast growth factor 5 (Fgf5) and insulin-like growth factor-binding protein 6 (Igfbp6), both of which are growth factors responsible for cell growth and survival. Hh signaling is recognized to be upregulated in pancreatic cancer stromal tissue. In pancreatic cancer cells lentiviral-mediated SMO suppression reduced cell proliferation and colony formation in vitro [64]. In vivo, SMO-silenced cells formed smaller xenograft tumors in nude mice. Using a parallel but complementary approach Singh et al. reported that treatment with SMO inhibitor vismodegib of human pancreatic cancer stem cells resulted in decreased cell viability and spheroid formation capability [65]. Though paracrine Hh signaling has long been associated with PDAC, Lee et al. report that Hh inhibition in pancreatic stroma results in increased tumor size and reduced overall animal survival [66]. In medulloblastoma (MB), Shh promotes hyperplasia of cerebellar granule precursor cells through the activation of HDACs, which in turn permit chromatin activity that leads to a sustained proliferative signal. Inhibiting the activity of HDACs with trichostatin A abrogated Shh-driven proliferation [67]. This study brought to light an interesting and relevant connection of activated Hh signaling with the chromatin-remodeling program. Furthermore, dysregulated Hh signaling upregulates levels of Cyclin D1 which directly leads to excessive proliferation as Cyclin D1 facilitates the ability of cells to bypass the mitotic cellular checkmarks leading to MB formation [68] and neuroblastoma [69]. As will be discussed in greater detail later, it was recently found that Hh signaling upregulates telomeric activity by increasing TERT expression in glioblastoma multiforme (GBM) [70]. The sustained proliferative signal leading to cancer development results from a combination of conditions such as upregulated growth factors and growth receptors, replicative immortality, and growth suppression evasion; those aspects will be discussed as their own specific hallmarks. The involvement of Hh signaling in proliferation, either pathologically or developmentally is evident from years of cancer research making Hh signaling a vital tool for cancers to employ.

### Replicative immortality

Cancer by definition is the uncontrolled replication of cells. Cancers successfully employ telomeric activation to maintain maximum replication; this is supported by the Hh pathway’s ability to upregulate telomerase activity. As the normal function of Hh signaling pathway is to promote embryonic development, it essentially controls and influences cellular division allowing its implication in various cancers as a driver for telomerase activity. The telomerase subunit is made up of telomerase reverse transcriptase (TERT) and telomerase RNA component, both of which make up the complex that drives cell replication [71]. Recently, it has been shown that GLI1 and GLI2 drive human TERT in colon and prostate cancers as well as GBM [70]. Blocking GLI activity by GANT-61 abrogated the enzymatic function of TERT. In addition, whole genome sequencing of SHH-driven MB showed a strong association with TERT promoter mutations [72]. Such robust influence on telomere function makes Hh signaling activation a valuable asset for tumor initiation.

### Deregulated cellular energetics

It is widely accepted that tumors adapt to hypoxic environments and survive by upregulating genes that promote anaerobic metabolism such as hypoxia-inducible factor-1α (HIF-1α) [73]; this is known as the “Warburg effect”. HIF-1α enables tumor cells to harness their energy by resorting to glycolytic instead of oxidative metabolism through up-regulating glucose transporters and pyruvate dehydrogenase kinase 1 (PDK1). PDK1 blocks the pyruvate dehydrogenase facilitated conversion of pyruvate to glucose, instead directing its conversion into lactate by lactate dehydrogenase A (LDHA). This leads to an increase in lactate production and consequently, increased glucose uptake [74]. Also, it is widely accepted that the lack of blood perfusion in hypoxic environments can hinder drug delivery and cause chemotherapy resistance in cancer [75–77]. Hh signaling drives hypoxia responses in different cancer models. In MB, Hh signaling is overly activated and drives glycolysis by upregulating hexokinase 2 and pyruvate kinase M2 that cumulatively promote lactate production [78]. Using a pyruvate kinase inhibitor reduced Hh-driven in vitro proliferation and in vivo MB growth. In PDAC, Hh pathway is non-classically activated by hypoxia, bypassing Hh ligand binding and Smo activation and directly activating Gli1 resulting in enhanced invasiveness through the induction of MMP2 and MMP9. These effects were abrogated upon blocking Gli1 [79, 80]. Additionally, mice with cervical cancer xenografts that were exposed to hypoxic conditions sustained higher levels of metastasis. When Hh signaling was blocked with 5E1 antibody, primary tumor growth and lymph node metastasis were reduced [81]. Collectively, this body of evidence supports the role for Hh signaling in promoting a tumor cell metabolism shift aiding in solid tumor progression, invasiveness, and therapy resistance.

### Inflammation

Cancer has long been associated with inflammatory responses [82, 83]. Inflammation in the tumor microenvironment drives several processes involved in the progression of cancer such as proliferation, EMT, angiogenesis, and metastasis through the upregulation of several pathways including NF-κB, TGF-β, and Hh signaling. Nucleotide-binding oligomerization domain-containing protein 2 (NOD2) signaling in inflammatory bowel disease is typically important for recognizing pathogenic patterns and promotes inflammation to elicit their removal by the immune system. NOD2 is reported to activate SHH and upregulate GLI1, further intensifying the inflammatory response [84]. The inhibition of Hh signaling in rats with arthritis using Smo inhibitor cyclopamine, reduced the expression of pro-inflammatory molecules such as TNF-α, IL-1β and IL-6 [85]. These pro-inflammatory molecules were also reduced in a mouse hepatocellular carcinoma model by vismodegib, which also targets Smo [86]. Patients with chronic cholecystitis often advanced to gallbladder carcinoma; and patients whose tumor samples expressed high levels of GLI1 were associated with unfavorable survival outcomes. In *Helicobacter pylori*-driven gastritis that progressed to gastric metaplasia, Gli1 was found to drive myeloid cell differentiation and upregulation of IL-6, IL-1β, and TNFα, three important cytokines for activating STAT3 signaling, the main promoter of the inflammatory response [87]. These effects were not observed in mice expressing mutant Gli1, directly implicating Hh signaling in gastric cancer initiation. Also in gastric cancer, tumor derived ligands behave as chemoattractants for macrophages that further intensify the inflammatory cascade leading to gastric cancer progression [88]. In gastric tissue specifically, Hh signaling has to be tightly regulated as it is vitally necessary for gastric tissue morphogenesis, however, its uncontrolled activation can contribute to the chronic inflammation associated with gastric cancer as mentioned above. As such, multiple evidences clearly show that Hh signaling is capable of promoting the inflammatory process in the microenvironment, which facilitates cancer initiation and progression.

### Angiogenesis

The formation of new limbs as development progresses requires the creation of blood vessels through the induction of pro-angiogenic factors such as vascular endothelial growth factors (VEGF) and fibroblast growth factors (FGF). Both VEGF and FGF are direct transcriptional targets of the Hh signaling pathway [89]. In an in vivo orthotopic hepatocellular carcinoma model, Smo inhibition using vismodegib downregulated VEGF expression leading to decreased blood vessel density and tumor growth [90]. In vitro however, vismodegib did not affect cell viability or migration, which is unsurprising considering the necessary complex physiological environment that allows for angiogenesis. Analysis of colorectal cancer patient specimens showed a strong positive correlation between Gli1 and VEGF-C and its receptor VEGFR3 [91]. In breast cancer, Hh signaling enhanced tumor angiogenesis independently of VEGF activation. SHH-mediated GLI1 activity lead to the transcriptional activation of the cysteine-rich angiogenic inducer 61 (CYR61); silencing CYR61 resulted in reduced tumor vasculature of SHH-driven xenografts [92]. A recent report in pancreatic cancer showed high expression of Hh co-receptors GAS1, BOC, and CDON in CAFs leading to larger tumor growths and increased vascularity. Deleting the three co-receptors blocked these tumorigenic properties and angiogenesis [93]. The implication of several members of the Hh pathway including the ligands, the receptor, and even co-receptor involvement convincingly demonstrates the crucial influence of Hh signaling on angiogenic changes leading to the establishment of vasculature necessary to support the growing tumors and facilitate metastasis.

Although the majority of published literature largely associates Hh signaling with increased angiogenesis, several reports have emerged in pancreatic cancer models showing increased vascularization enhancing drug delivery upon the inhibition of paracrine Hh signaling in pancreatic stroma using SMO inhibitors [94–96]. Thus, more thorough investigation of Hh activation and its effect on angiogenesis is necessary.

### Evading immunity

**Fig. 3:**
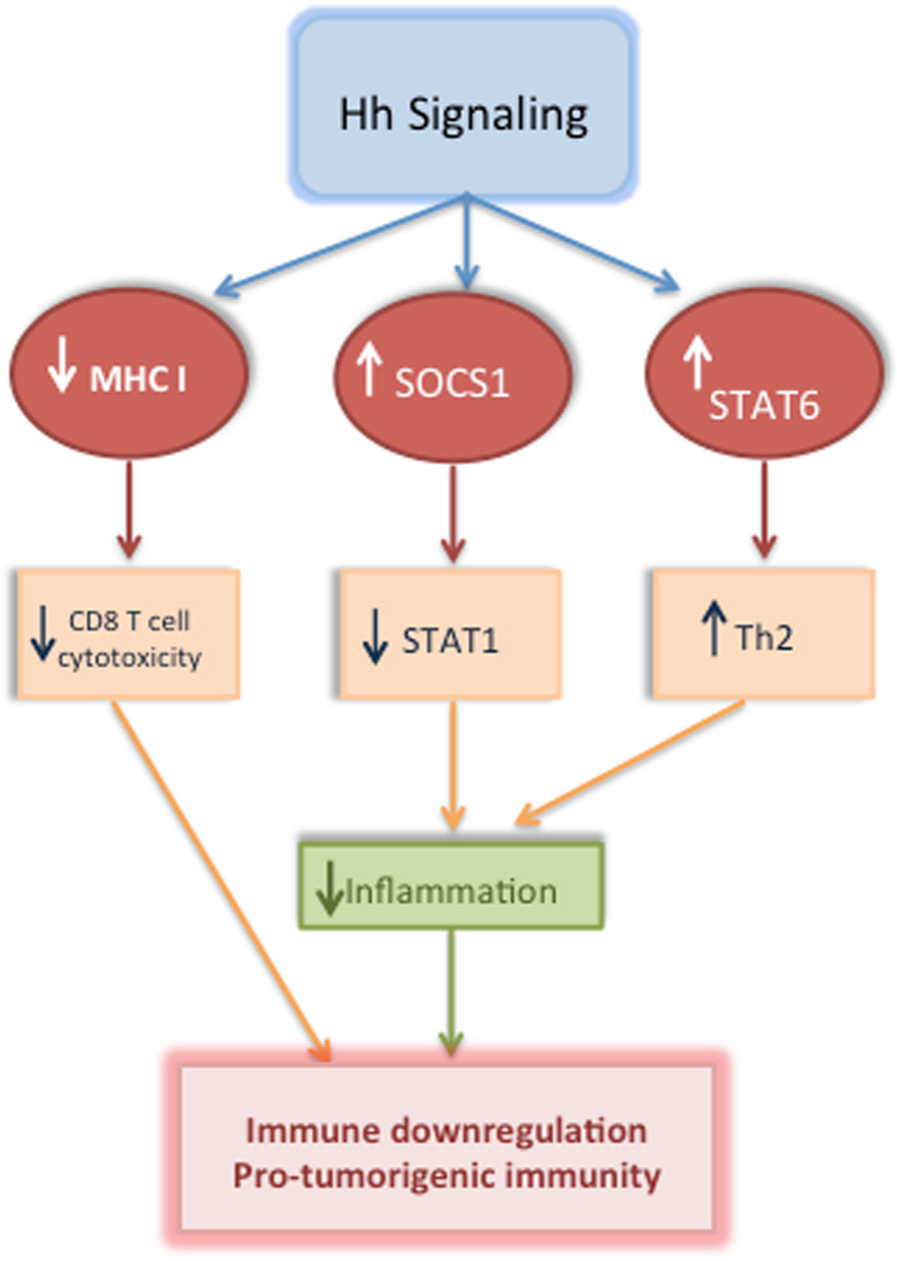
Emerging preliminary evidence implicates Hh signaling activation in immune system suppression as a result of the pathway’s ability to downregulate MHC I which suppresses cytotoxic T cell tumor cell clearance, upregulate SOCS1 which inhibits STAT1 signaling, and upregulate STAT6 which promotes T cell polarization toward the Th2 phenotype. All three properties result in diminished immune system function against tumors and the promotion of pro-tumorigenic functions by T cells

Although the human immune system is fully capable of eliminating foreign matter and abnormal cells, cancer cells evade the immune system through various mechanisms [97]. Cancer cells can prevent T cell proliferation via upregulating toll like receptors [98], downregulating the MHC molecules and antigens presented on their surface [99], and immunoediting immune cells in their microenvironment to promote cancer growth and survival. This involves activating regulatory T cells and myeloid-derived suppressor cells (MDSCs), which suppress anti-tumor immunity [100]. In recent years, several groups have reported an emerging link between Hh signaling and the ability of tumors to evade the immune system (Fig. 3). In patients with BCC that were treated with vismodegib, there was CD4 and CD8 T cell infiltration into the tumor with concomitant upregulation of MHC class I molecules [101]. In T cells, Hh ligands activated IL-4, a cytokine responsible for skewing T cells toward the Th2 phenotype. This T cell sub-type enhances allergy reactions and suppresses immune system surveillance thereby promoting tumor growth [102]. JAK-STAT signaling is an extensive network of molecules involved in developmental and reparative functions such as cell proliferation, migration, and immune system properties. IFNγ-activated STAT1 signaling induces apoptosis, halts progression through cell cycle and boosts anti-tumor immunity. This signaling pathway is negatively regulated by the suppressor of cytokine signaling (SOCS) family of nine proteins. In keratinocytes and MB cells, SOCS1 was found to be a direct target of GLI2, blocking the IFN-γ/STAT1 pathway and inhibiting cell cycle arrest and downregulating anti-tumor immunity [103]. Studies in a GLI2 transgenic mouse showed significant reduction of T cell activation which gave rise to impaired NF-κβ signaling interfering with its normal function of activating a pro-inflammatory immune cascade [104]. Though Hh signaling seems to be connected to impaired T cell activation, some reports confirm Hh activation is linked cytotoxic T cell activation; this T cell-induced cytotoxicity was abolished upon using SMO and GLI inhibitors [105]. This dual activity is unsurprising for Hh signaling, as it is important in T cell development [106], however, in the presence of pathological conditions, the functions of the pathway could shift. While the effects of Hh signaling on macrophages are unknown, Hh signaling is evidently involved in suppressing host immunity to promote tumor growth by activating T regs, MDSCs, and the Th2 phenotype that promote angiogenesis and extracellular matrix remodeling.

### Resisting cell death

One of the most important mechanisms by which cancers support their growth and immune system evasion is through avoiding apoptosis [107, 108]. Apoptosis is the programmed, self-destruction of cells that develop structural damage beyond the capacity of DNA repair machinery. The apoptotic pathway involves a family of caspase proteins that lead to the formation of the apoptosome; the BCL2 family of proteins inhibits this process. In MM-derived cells, the tumor cells secrete SHH; autocrine SHH signaling induces cell proliferation, chemotherapy resistance, and protection against apoptosis by upregulating BCL2 [109]. Neutralizing antibodies against SHH sensitized MM cells to apoptosis. RNA analysis of clinical samples of GBM revealed upregulated expression of GLI1 and PTCH1. vismodegib treatment reduced GLI1 expression concomitant with the induction of apoptosis and cell cycle arrest [110]. AKT (protein kinase B) promotes cell growth and survival by inhibiting the pro-apoptotic function of the BAD protein and inducing the NF-κβ pro-survival pathway. In acute T-cell leukemia, GLI inhibition via GANT-58 induced apoptosis and AKT-driven anti-tumor responses [111]. In AML, GLI1 is overexpressed and is considered a negative prognostic marker; its inhibition reduced cell proliferation and induced apoptosis [112]. In hepatocellular carcinoma, Hh signaling enhances cell proliferation and therapy resistance. Inhibition of Smo via cyclopamine in addition to radiation reduced cell survival and tumor size and induced DNA double stranded breaks (DSB) [113]. By virtue of Hh signaling, abnormal cancerous cells override death signals and continue to grow uninterrupted, further enabling establishment of the tumor mass.

### Activating invasion and metastasis

The ability of tumor cells to metastasize is permitted through the flexibility of the cells to alter their shape and function, break through the basement membrane, extravasate into lymph and blood vessels, and travel to distant locations where they occupy a distinct microenvironment [114, 115]. Several of these abilities are modulated through the Hh pathway. SHH is highly correlated with aggressive disease stage in retinoblastoma, progressively increasing as the disease advances to metastasis [116]. In a renal cell carcinoma xenograft model, Smo antagonism with Erismodegib inhibited proliferation, migration, invasive properties, and lung metastasis [117]. In ER-positive breast cancers, Gli1 promotes cancer stem cell (CSC)-like properties enhancing invasion and EMT [118]. Also in breast cancer, IHH-induced GLI1 activity promoted osteoblast expression of Rank ligand and osteopontin (OPN). Both are vital molecules involved in osteoclast activity, enabling dysregulated bone remodeling that creates an osteolytic, pro-metastatic niche in the bone tissue [119]. A similar increase of GLI1-induced OPN was seen in human melanoma; an effect that was abolished with SMO antagonist administration [120]. Hh signaling activation in thyroid CSCs was associated with elevated expression of Gli1 and Snail and consequently stimulated EMT associated with high numbers of CSCs. Blocking Hh signaling induced the radiation sensitivity of the tumor cells and decreased their self-renewal activity [121]. A network modeling exercise study to determine TGF-β–induced networks in several murine and human hepatocellular cancer lines, revealed that TGF-β induced EMT and lead to coupled activation of the Hh and Wnt signaling pathways, further creating a network for persistence of the EMT phenotype [122]. As a developmental pathway, Hh signaling greatly influences cellular differentiation and proliferation, therefore its potential in promoting EMT is unsurprising. As such, Hh signaling profoundly enhances the tumor cells’ ability to change their phenotype and upregulate matrix proteases [90, 91] cumulatively facilitating their movement and expansion resulting in enriched invasive potential.

*Hh signaling and cancer stem cells:* Given the role of Hh signaling during development when there is a dynamic shift between cellular phenotypes from epithelial and mesenchymal type, it comes as no surprise that this is recapitulated in cancer progression [123]. Staining of gastric cancer patient samples and patient cell line derived-xenografts showed that the CD44-expressing population of stem-like cells overexpresses Hh proteins; consequently, blocking Hh signaling with SMO inhibitor vismodegib reduced the colony formation ability of these cells [124]. A more recent report again implicates SHH/GLI1 activation in gastric CSCs drug resistance; a function reversed upon GLI and SMO inhibition by GANT61 and vismodegib treatments respectively [125]. Likewise, in colon cancer, SMO antagonist cyclopamine reduced stem cell marker expression and EMT in cell line spheroidal culture [126]. p63, a key regulator in cell differentiation has been shown to induce stem cell-like properties. In a breast cancer model, tumor cell growth and self-renewal properties were modulated by p63-driven induction of Shh, Gli2, and Ptch expression [37]. Also in pancreatic cancer, Hh activity is strongly correlated with stem-like properties. The novel approach of sulforaphane administration, a cruciferous plant derivative shown to reduce inhibit stem cell viability, inhibited SHH-driven activation and reduced the expression of stem-cell pluripotency markers, Nanog and Oct-4, and functionally reduced spheroid formation [127]. Also in pancreatic cancer, blocking SHH signaling with vismodegib or Gli1 and Gli2 silencing reduced the viability of stem-like cells, decreased spheroid formation and induced apoptosis [66]. The involvement of Hh signaling in CSC sustenance promotes cancer resistance to therapies through the induction of this stem-like state, which are largely quiescent during the initial chemotherapy treatments, then reactivating a recurring disease and potential metastasis.

### Genomic instability

The progression of cells through the cell cycle in order to undergo replication requires the successful advancement through many checkpoints which would suppress growth if cells are experiencing chemical and physical stress or DNA damage. Cancer cells though defective in structure and function, employ different signaling pathways, including the Hh pathway, to bypass those regulatory checkpoints [128]. Aberrant Ptch1 and Sufu mutations in BCC associate with abnormal cell cycle entry, cyclin D expression, increased DNA damage, and impaired p53 expression. In these cases cell cycling due to loss of cell cycle arrest and progress through the G2/M checkpoint occurred independently of p53 [129]. Recently, aberrant expression of Gli1 in tumor cells showed to regulate tumor cell response to replication blocks [130]. In these studies, Gli1 expression has been shown to suppress replication stress in tumor cells by regulating ATR mediated Chk1 phosphorylation through transcriptional regulation of Bid. While Inhibition of Gli1 in tumor cells reduced Bid levels and its association with RPA-ATRIP complex leading to abrogation of ATR-mediated Chk1 phosphorylation. Thus, inhibition of GLI1 in tumor cells sensitized them to chemotherapeutic agents that target DNA Topoisomerase I [130]. A novel finding in MB implicates upregulation of the co-receptor Boc in aberrant Hh activation in concert with Cyclin D1 and N-Myc causing granule cell precursors to overproliferate and lead to MB [68]. In keratinocytes, the GLI2-β isoform, which lacks the N-terminal repressor domain, was found to induce structural chromosomal abnormalities such as chromosomal translocation, aneuploidy, and tetraploidy; the overexpression of this N-terminal domain abolished apoptosis and cell cycle checkpoint by decreasing 14-3-3s (transcriptional target of p53) and CDK inhibitor p21 [131]. The capability of Hh signaling to bypass cell cycle checkpoints maintains a constant activation in cellular replicative programming, allowing even abnormal cells that would normally cease to replicate to continue unrestricted division and expansion.

### Evading growth suppression

A possible mechanism by which tumors can evade cell growth suppression other than cell cycle checkpoints is their ability to regulate autophagy. Autophagy is the process by which cells get degraded and their components recycled as a result of cellular stress or infections [132]. In stressful conditions such as hypoxia, mitochondrial autophagy is induced by the activation of BNIP3 protein (BCL2 and adenovirus E1B 19 kDa-interacting protein 3) and its induction of autophagosome formation. It can also result in cytochrome c and caspase activation leading to apoptosis. Hh signaling has been shown to downregulate this process in several biological systems including cancer. Holla and colleagues have shown that microorganisms including Mycobacteria, Shigella, and Listeria downregulate the body’s autophagy response by activating the Hh signaling pathway [133]. In non-small cell lung cancer (NSCLC), blocking SMO and GLI1 induced autophagy and apoptotic responses [134]. Furthermore, in pancreatic ductal adenocarcinoma (PDAC) cells, blocking the activity of Gli proteins using GANT-61 induced cell autophagy and reduced cell viability [135]. Similar responses were seen in hepatocellular carcinoma cells using GANT-61 characterized by upregulation of Bnip3 [136]. Autophagy was the result of Bnip3-driven displacement of the anti-apoptotic Bcl-2 from Beclin-1, the driver of autophagy. In chondrosarcoma, Ihh expression is upregulated and associated with increased Gli activity. Inhibition of Gli1 suppressed cell proliferation through promoting cell cycle arrest, induced apoptosis by downregulating Bcl2, and altered mTOR phosphorylation culminating in autophagy [137]. Avoiding the normal suppressive signals that eliminate infected or abnormal cells by virtue of dysregulated Hh signaling promotes tumor cell growth and enhances the potential to acquire and accumulate additional tumor promoting mutations.

### Hh signaling and therapy resistance – an important feature of cancer recurrence

In addition to the impact of Hh signaling on profoundly influencing several cancer processes, it also desensitizes cancer to therapies. In breast cancer, the OPN oncoprotein was shown to activate non-classical Hh signaling and GLI transcription, resulting in the upregulation of multidrug resistance genes viz. ATP-binding cassette transporters (ABCB1) and ATP-binding cassette sub-family G member 2 (ABCG2). Treatment of cells with GLI inhibitor GANT-61 or silencing GLI1 expression abrogated this effect [138]. In prostate cancer, SHH signaling promoted resistance to paclitaxel through the overexpression of ABCB1 [139]. In castration-resistant prostate cancer, patients develop resistance against the normally used docetaxel treatment regimen. Combining vismodegib Hh inhibitor with docetaxel significantly reduced prostate cell proliferation, migration, and induced apoptosis in vitro and in patient xenografts [140]. In a GBM model, miR-9-mediated downregulation of PTCH1 resulted in the upregulation of Hh signaling and resistance to the standard-of-care drug, temozolomide that was reversed by treatment with the Smo inhibitor, vismodegib [141]. Although vismodegib is generally effective in BCC treatment, SMO point mutations can prevent vismodegib binding thus giving rise to drug resistance [142]. The formulation of a novel dual compound NL-103 targeting both, mutant SMO and histone deacetylases (HDAC) overcame vismodegib resistance. By virtue of inhibiting HDACs, NL-103 downregulated Smo and Gli2 [143]. Additionally in MM, SHH signaling promoted resistance to chemotherapy-induced apoptosis, which was reversed with the SHH blocking antibody 5E1. In ovarian cancer, combining proteasome inhibitors with Hh antagonist promoted paclitaxel sensitivity by reducing drug resistance gene ABCB1 and promoting apoptosis [144]. Erlotinib, an epidermal growth factor receptor-tyrosine kinase inhibitor, is used for the treatment of NSCLC, and patients often develop resistance against it through inducing an EMT phenotype. Inhibiting Hh signaling using vismodegib, sensitized NSCLC cells to Erlotinib [145]. Hh signaling is strongly linked to chemotherapy resistance in several cancer types as mentioned above; therefore combination therapy using Hh inhibitors in addition to standard of care cytotoxic chemotherapies will greatly improve patient response and potentially significantly enhance survival.

Hh signaling also desensitizes tumor cells to radiotherapy. In hepatocellular carcinoma, SHH-driven signaling induced PTCH1 and Gli1 expression; this protected cells against ionizing radiation (IR) [146]. The use of an antibody to neutralize SHH ligands or a GLI1 silencing approach reversed this protective capability. In GBM, Shh signaling was activated upon IR treatment [147]. Additionally, cyclopamine treatment increased DNA DSB as indicated by the induction of H2AX, a histone family member that becomes phosphorylated upon DNA breaks. In colon cancer, radiation therapy resistance was abolished upon treating cells with GLI inhibitor GANT-61 concomitant with detection of DNA aberrations as indicated by induced H2AX and apoptosis as indicated by caspase 3 [148]. Therefore, there is direct evidence for the involvement of Hh activation and protection against DSB; effects that are reversed with Hh blockade at different stages: antibody ligand blocking, Smo inhibition, or Gli1 silencing.

## Conclusion

It is evident from the presented compilation of literature that the Hh signaling pathway is extensively studied and significantly linked to several cancer types. Aberrations in almost all molecular members of the pathway have been reported across multiple malignancies. The pathway’s ability to promote replicative immortality, evade genomic instability and growth suppression, sustain proliferative signaling, and switch cellular energetics are crucial for the tumor cells’ ability to grow uncontrollably, avoiding multiple levels of homeostatic cellular regulation that prevent damaged cells from surviving. As the tumors establish in their niche, they employ Hh signaling to induce angiogenesis, take advantage of tumor promoting inflammation and escape immune system surveillance to facilitate tissue invasion and metastasis.

In closing, Hh signaling is activated across multiple cancer types in many different ways: through mutations or through non-classical ligand-independent or through non-canonical modes. Hh signaling influences every aspect of a tumor cell: initiation, establishment and its metastatic journey. With the promising success of inhibitors of this pathway in the clinic, the pathway continues to offer the scope to direct and design inhibitors to function at multiple levels. At present, a large cadre of chemically modified pharmacological inhibitors has been developed to target SMO. In order to pursue the elimination of the rapidly-evolving tumor and its associated milieu, it is necessary to continue efforts to target this pathway at all levels. Avenues to target and block the activity of ligands, block co-receptors Boc, Cdo, and Gas1 and interfere with the activity of GLI transcription factors are currently being explored by many laboratories. Discovered in the *Derris glabrescens* plant, Glabrescione B is among the newly identified, naturally occurring small molecule inhibitor, functioning in a similar mechanism to GLI inhibitors GANT-58 and 61 [149]. The recent characterization that the bromodomain and extra terminal domain (BET) member BRD4 protein can bring about activation of Hh/GLI signaling by upregulating the expression of GLI proteins, has brought to the forefront the promising use of the small molecule BRD4 inhibitor JQ1, to abolish dysregulated signaling in tumors that bear oncogenic SMO mutations [150]. With the knowledge of non-canonical Hh signaling, efforts need to be directed towards the discovery and development of small molecules that can interfere in this process in order to effectively eliminate resistant populations.

## Abbreviations

ABCB1: ATP-binding cassette transporters
ABCG2: ATP-binding cassette sub-family G member 2
AKT: Protein kinase B
ATR: Ataxia telangiectasia and Rad3-related protein
BAD: Bcl-2-associated death promoter
BCC: Basal cell carcinoma
BCL: B-cell CLL/lymphoma
BNIP3: BCL2/adenovirus E1B 19 kDa protein-interacting protein 3
BOC: Brother of CDO
CAF: Cancer-associated fibroblasts
CD: Cluster of differentiation
CDON: Cell adhesion molecule-related/downregulated by oncogenes
CHK1: Checkpoint kinase 1
CSC: Cancer stem cells
CYR-61: Cysteine-rich angiogenic inducer 61
DHH: Desert hedgehog
DSB: DNA double strand breaks
EMT: Epithelial-mesenchymal transition
ERK: Extracellular-signal-regulated kinases
FGF: Fibroblast growth factor
GANT: Gli antagonist
GAS1: Growth arrest-specific 1
GBM: Glioblastoma multiforme
GLI: Glioma-associated oncogene homolog
GPI: Glycosylphosphatidylinisotol
H2AX: H2A histone family, member X
HDAC: Histone deacetylases
Hh: Hedgehog
HIF: Hypoxia inducible factor
IFN-γ: Interferon gamma
IHH: Indian hedgehog
IL: Interleukin
JAK: Janus kinase
LDHA: Lactate dehydrogenase A
MB: Medulloblastoma
MDSC: Myeloid-derived suppressor cells
MM: Multiple myeloma
MMP: Matrix metalloproteinase
NF-κB: Nuclear factor –kappa beta
NOD2: Nucleotide-binding oligomerization domain-containing protein 2
NSCLC: Non-small cell lung cancer
OPN: Osteopontin
PDAC: Pancreatic ductal adenocarcinoma
PDK1: Pyruvate dehydrogenase kinase 1
PI3K: Phosphatidylinositol-4,5-bisphosphate 3-kinase
PTCH: Patched
RAS: Rat sarcoma
SHH: Sonic hedgehog
SMO: Smoothened
SOCS: Suppressor of cytokine signaling
STAT: Signal transducer and activator of transcription
SUFU: Suppressor of fused homolog
Th: Helper T cells
T regs: Regulatory T cells
TERT: Telomerase reverse transcriptase
TGF-β: Transforming growth factor beta 1
TNF: Tumor necrosis factor
VEGF: Vascular endothelial growth factor
VEGFR: Vascular endothelial growth factor receptor
