# Erratum to**: ‘**Hedgehog signaling: modulation of cancer properties and tumor microenvironment**’**

**DOI:** 10.1186/s12943-016-0522-6

**Published:** 2016-05-11

**Authors:** Ann Hanna, Lalita A. Shevde

**Affiliations:** Department of Pathology and Comprehensive Cancer Center, The University of Alabama at Birmingham, Birmingham, USA

Unfortunately, the original version of this article [1] contained errors. The words “properties” and microenvironment” have been spelt incorrectly in the title. The title should read as follows:

Hedgehog signaling: modulation of cancer properties and tumor microenvironment
